# Cardiomyocyte Overexpression of FABP4 Aggravates Pressure Overload-Induced Heart Hypertrophy

**DOI:** 10.1371/journal.pone.0157372

**Published:** 2016-06-13

**Authors:** Ji Zhang, Congzhen Qiao, Lin Chang, Yanhong Guo, Yanbo Fan, Luis Villacorta, Y. Eugene Chen, Jifeng Zhang

**Affiliations:** Department of Internal Medicine, Cardiovascular Center, University of Michigan Medical Center, Ann Arbor, Michigan, United States of America; Nagoya University, JAPAN

## Abstract

Fatty acid binding protein 4 (FABP4) is a member of the intracellular lipid-binding protein family, responsible for the transportation of fatty acids. It is considered to express mainly in adipose tissues, and be strongly associated with inflammation, obesity, diabetes and cardiovasculardiseases. Here we report that FABP4 is also expressed in cardiomyocytes and plays an important role in regulating heart function under pressure overload. We generated heart-specific transgenic FABP4 (FABP4-TG) mice using α myosin-heavy chain (α-MHC) promoter and human FABP4 sequence, resulting in over-expression of FABP4 in cardiomyocytes. The FABP4-TG mice displayed normal cardiac morphology and contractile function. When they were subjected to the transverse aorta constriction (TAC) procedure, the FABP4-TG mice developed more cardiac hypertrophy correlated with significantly increased ERK phosphorylation, compared with wild type controls. FABP4 over-expression in cardiomyocytes activated phosphor-ERK signal and up-regulate the expression of cardiac hypertrophic marker genes. Conversely, FABP4 induced phosphor-ERK signal and hypertrophic gene expressions can be markedly inhibited by an ERK inhibitor PD098059 as well as the FABP4 inhibitor BMS309403. These results suggest that FABP4 over-expression in cardiomyocytes can aggravate the development of cardiac hypertrophy through the activation of ERK signal pathway.

## Introduction

Cardiac hypertrophy, the thickening of heart muscle, is a compensatory response to physical stimuli or pathological insults in heart. During hypertrophy development, the energy expenditure and the structure of cardiomyocyte will change, and heart will utilize glucose instead of fatty acids as the main source of energy. These processes are mediated by stress-responsive and other signaling pathways [1–6]. Initially, these compensatory changes in heart can be adaptive, but sustained pathologic hypertrophy will eventually break the balance in heart metabolism and cause de-compensation, chronically leads to cardiac diastolic dysfunction or even heart failure, causing large number of morbidity and mortality in the world [7, 8].

Lipids and lipid related proteins are critical in the heart metabolism and play significant parts in cardiac hypertrophy development. For example, both over-expressing [9] and knock-out PPARγ[10] in mice heart can induce lipid disorder and leads to cardiac hypertrophy. Fatty acid binding proteins (FABPs) are a family of transport proteins with high affinity to long-chain fatty acids [11]. With an average size of 14-15-kDa and wide-range tissue expressions, these lipid chaperones transport cellular lipids and are actively involved in lipid droplets accumulation, endoplasmic reticulum signaling and membrane synthesis. Mitochondria and peroxisome also depend on FABPs for fatty acid transportation and oxidation [12]. Compared with other FABPs, the adipocyte FABP, commonly known as FABP4 or AP2 (Adipocyte Protein 2), is highly expressed in adipocyte and has multiple functions other than fatty acid transportation.

FABP4 is a direct target gene of PPARγ, and has been shown to activate multiple metabolic and inflammatory signal pathways in both adipocytes and macrophages [13], thus plays a critical role in insulin resistance [14], type 2 diabetes [15, 16], atherosclerosis and other metabolic syndromes [17]. Previous studies also indicated that FABP4 exists in heart [18]. Patients with metabolic syndrome had significantly increased FABP4 expression in epicardial fat [19–21]. FABP4 expression is relatively higher in capillary endothelial cells in mouse and human heart [18]. However, the role of endogenous FABP4 in heart, especially in cardiomyocytes has never been investigated. We hypothesize that FABP4 exist in cardiomyocytes and that high level of cardiac FABP cause damage directly to cardiomyocytes. Considering the large number of diabetes patients and the popularity of PPARγ agonists (Rosiglitazone, Pioglitazone) class anti-diabetes drugs, elevated cardiac FABP4 expressions may be quite common among metabolic syndromes patients. Thus, it is of clinical significance to investigate the role of FABP4 in cardiomyocytes. Consistent with our hypothesis, recent in vitro experiments demonstrated that exogenous FABP4 could strongly suppress the shortening amplitude of cardiomyocytes, suggesting that FABP4 is more than a marker to cardiac dysfunction [22, 23].

To characterize the direct impact of cardiac FABP4, we generated a transgenic mouse model that specifically over-expressing the human FABP4 gene in cardiomyocytes by using the α-MHC promoter [24]. We report that such animal is sensitive to heart pressure overload and will develop more cardiac hypertrophy after TAC than WT littermate controls. Thus, we reveal that FABP4 is a positive regulator of cardiac hypertrophy. To the best of our knowledge, these findings disclose for the first time a novel role for FABP4 in cardiomyocytes and cardiac hypertrophy.

## Materials and Methods

### Animal Model

Cardiomyocyte-specific FABP4 transgenic (FABP4-TG) mice were generated by injection a construct composed of a mouse α-myosin heavy chain promoter (Chr.14, GRCm38.p2: 54965464–54970938, 5.4kb) and human FABP4 coding sequence (NM_001442.2) followed by human growth hormone polyadenylation signal (HGH-pA). The microinjection and embryo transfer were performed by Transgenic Animal Model Core at the University of Michigan. Animals had free access to normal rodent chow diet and water. Transgenic mice were maintained by breeding with wild-type (WT) C57BL/6 mice. Ten-week-old male FABP4-TG and WT littermate control mice were subjected to transverse aortic constriction (TAC) or a sham operation [25, 26]. In brief, all animals were anesthetized by intraperitoneal injections of ketamine (100mg/kg body weight) and xylazine (5mg/kg body weight). Anesthetized mice were placed on a heating pad (37°C) during surgery to maintain body temperature. Hair was removed from thoracic area with depilatory cream. Ophthalmic ointment was used for sterile prep which contains at least 3 alternating passages of scrub and rinse. We then made a horizontal incision (1-3cm length) near the suprasternal notch while keeping the pleural space intact to make sure the animal can breathe normally. The transverse aorta between the right innominate and left carotid arteries was carefully banded to the diameter of a 27-gauge needle using a 7–0 silk suture. For sham group, same procedures were operated on sex- and age- matched mice, only the actual aortic binding was omitted. After surgery, mice were treated with buprenorphine or carprofen once pre-emptively, then for a minimum of 24 hours post-operation, then as needed for pain relief. All animals were monitored on a daily basis. Fourteen days after surgery, the mice were sacrificed and the hearts were harvested. All experimental procedures were approved by the Institutional Animal Care and Use Committee of the University of Michigan (Protocol number #PRO00006176).

### Histology

Hearts were harvested from FABP4-TG and WT control mice that underwent a TAC or sham operation and fixed with 4% formaldehyde overnight. Heart samples were dehydrated and embedded in paraffin wax following standard laboratory procedures. Serial horizontal paraffin heart sections (5 μm) were mounted on glass slides and deparaffinized. Sections were then stained with hematoxylin and eosin for histopathological examination under microscopy. Cardiac fibrosis was determined by Sirius Red staining as described previously [26]. For cardiomyocyte cross-sectional area determination, 50 individual cells per slide were determined in the images and the cell area sizes were calculated by computerized pixel counting.

### Echocardiogram assay

The echocardiographic assessment was performed by the Echocardiography Core in the University of Michigan following standard protocols [27]. In general, all mice were anesthetized by 3% isoflurane and were placed on a warming pad to maintain body temperature. Additional 1–2% isoflurane was supplied *via* a nose cone to maintain a surgical plane of anesthesia. The hair was removed from the upper abdominal and thoracic area with depilatory cream. Electrocardiogram (ECG) was monitored *via* non-invasive resting ECG electrodes. Transthoracic echocardiography was performed in the supine or left lateral position. M-mode, Doppler and tissue Doppler echocardiographic images were recorded using a Visual Sonics’ Vevo 770 high resolution *in vivo* micro-imaging system. Systolic and diastolic dimensions and wall thickness were measured in M-mode in the parasternal short axis view at the level of the papillary muscles. Fractional shortening and ejection fraction were calculated from the M-mode parasternal short axis view. M-mode echocardiographic images were recorded using a GE S10 MHz phased-array transducer, connected to a General Electric, Vivid 7 Ultrasound System.

### Cell culture

Primary neonatal rat cardiomyocytes were enzymatically isolated from 1- to 2-day-old Sprague-Dawley (SD) rats using a neonatal cardiomyocyte isolation kit (Worthington Biochemical Corporation). Cardiomyocytes were plated at a density of 1.0 × 10^6^ cells/well in six-well plates and cultured in DMEM medium containing 10% fetal bovine serum. Adult mice cardiomyocytes were isolated from 8-week-old WT and FABP4-TG mice following previous published protocols [28]. Recombinant adenovirus for FABP4 over-expression and its respective control virus Ad-GFP were generated as described previously [26]. Ad-GFP and Ad-FABP4 infections were performed at a multiplicity of infection (MOI) of 5. The FABP4 expression level was detected by Western blotting. For reagents used in cellular experiments, Angiotensin II and ERK inhibitor PD098059 were purchased from Sigma-Aldrich. The FABP4 inhibitor BMS309403 and FABP4-KO mice heart protein samples were generous gifts from Dr. Aimin Xu from the University of Hong Kong.

### Western blotting

Heart protein lysates and cellular protein lysates were prepared using T-PER tissue protein extraction buffer and M-PER mammalian protein extraction buffer (Thermo Scientific), respectively. Rabbit anti-phosphor-ERK, goat anti-total ERK, goat anti-TNF-α and goat anti-GAPDH antibodies were purchased from Santa Cruz Biotechnology, whereas rabbit anti-phosphor-AKT and anti-GSK3β antibodies were purchased from Cell Signaling Technology. The rabbit anti-FABP4 from Cayman and the rabbit anti-Troponin I from Abcam were used in Immunofluorescence and Western blots.

### Quantitative real-time PCR analysis

Total heart RNA and cellular RNA were isolated using Trizol Reagent from Invitrogen. cDNA was synthesized from 1μg RNA from each sample using SuperScript III reverse transcriptase (Invitrogen). A SYBR Green Supermix (Bio-Rad) was used for quantitative real-time PCR (qRT-PCR).

### Statistical analysis

Data are presented as mean±SD. Differences between mean values were evaluated by Student’s t-test or one-way ANOVA, with values of p<0.05 indicating a significant difference.

## Results

### FABP4 expresses in cardiomyocytes and can be induced by PPARγ

**Fig 1:**
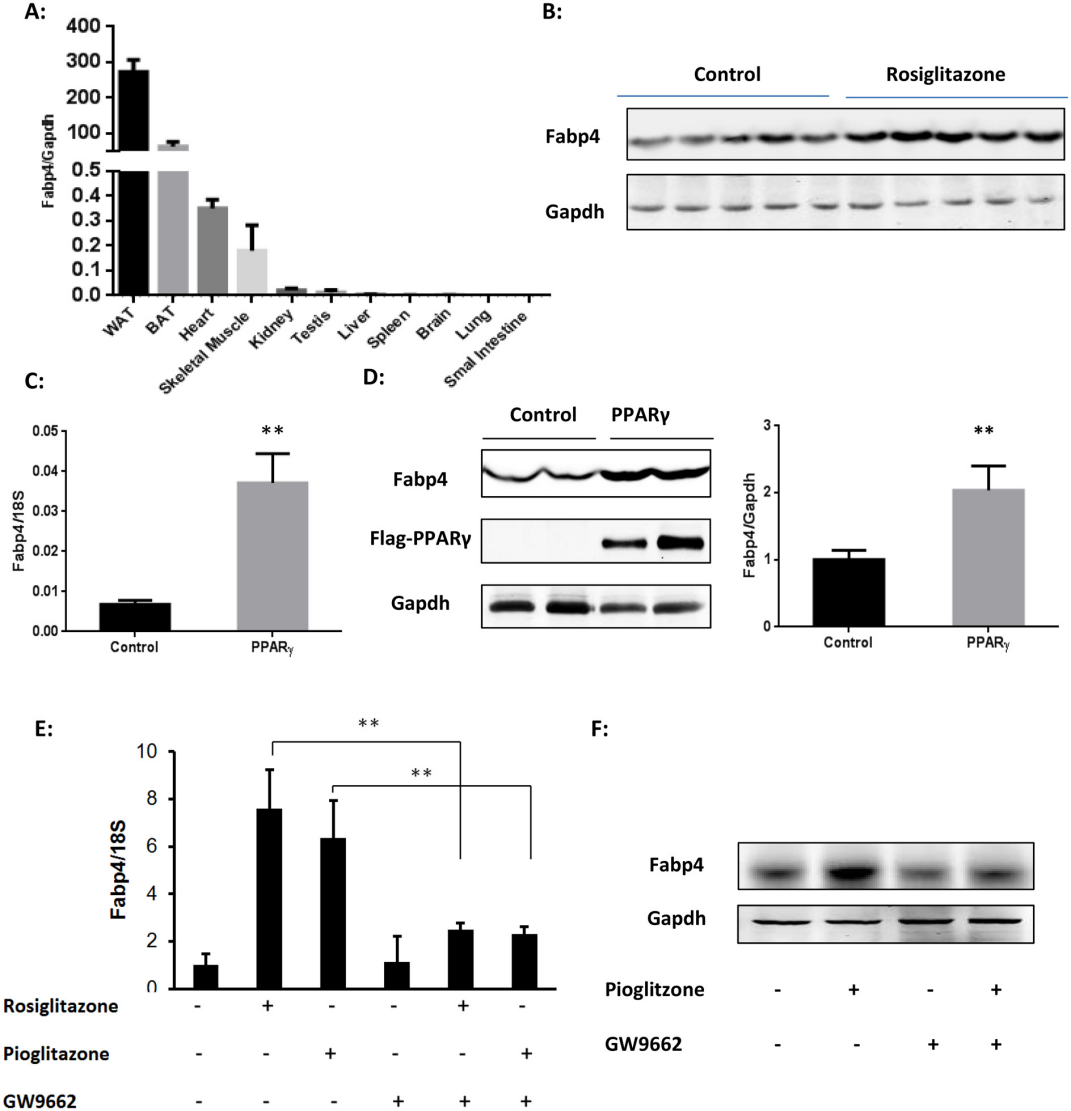
PPARγ induces FABP4 expression in heart. (A) Tissue distribution of FABP4 levels in mice was determined by qRT-PCR (mean±SD, n = 3 in each group). (B) Representative western blots showing increased expression of FABP4 protein in heart from Rosiglitazone treated C57BL/6 mice (5 mg/kg body weight per day by gavage for 14 days) compared with placebo group. (C&D) Over-expression of PPARγ in NRCMs increased the level of FABP4 mRNA (C) and protein (D). Values for the specific mRNA levels normalized by 18S rRNA are expressed as mean±SD (n = 4, **p<0.01); Representative western blot using GAPDH as the internal control. Relative expression levels of FABP4 protein normalized by GAPDH are expressed as mean±SD (n = 4, **p<0.01) and shown in the right panel. (E) qRT-PCR was used to measure FABP4 expression level changes in NRCM treated with PPARγ agonists for 24 hours (Rosi: 1μM, Pio: 10μM, GW9662: 10μM). Values for the specific mRNA levels normalized by 18S rRNA are expressed as mean±SD (n = 4, **p<0.01); (F) FABP4 protein level in NRCM treated with PPAR γ agonists was measured by Western blots.

FABP4 is expressed constitutively in a variety of organs, including the heart. By qRT-PCR, we determined mouse FABP4 tissue distribution and found that white fat has the highest expression level of FABP4 among different tissues, followed by the brown fat. In non-adipose tissue, heart has the highest expression level of FABP4 (Fig 1A). Consistent with the previous study identifying FABP4 as a direct target gene of PPARγ [29–31], the cardiac FABP4 expression can be strongly induced by PPAR γ activation. As shown in Fig 1B, the FABP4 expression level in heart has significantly increased after mice were fed with Rosiglitazone for 2 weeks.

To determine whether FABP4 is expressed in cardiomyocytes, we first cultured neonatal rat cardiac myocytes (NRCMs) and transfected a Flag-tagged-PPARγ expression plasmid into NRCMs to test if FABP4 expression can be detected in cardiomyocytes and further regulated by over-expressing PPARγ. RNA and protein were extracted from NRCMs 48 hours after transfection. The expression of FABP4 was markedly upregulated by PPARγ over-expression as assayed by qRT-PCR (Fig 1C) and Western blotting (Fig 1D). Similarly, PPARγ agonists can also induce FABP4 in NRCMs, and this induction can be abolished by PPARγ specific inhibitor GW9662 compound [32, 33] (Fig 1E and 1F). Immunofluorescence experiments also confirmed the existence of FABP4 in cardiomyocytes and the induction of FABP4 by PPARγ agonist (Figure A in S1 Fig). Notably, cardiomyocytes has a much higher level of FABP4 expression than cardiac fibroblast (Figure B in S1 Fig). These results indicate that FABP4 is expressed in cardiomyocytes and can be regulated by PPARγ, suggesting FABP4 may have potential important roles in cardiac metabolism and functions.

### FABP4 over-expression in cardiomyocyte aggravates cardiac hypertrophy

**Fig 2:**
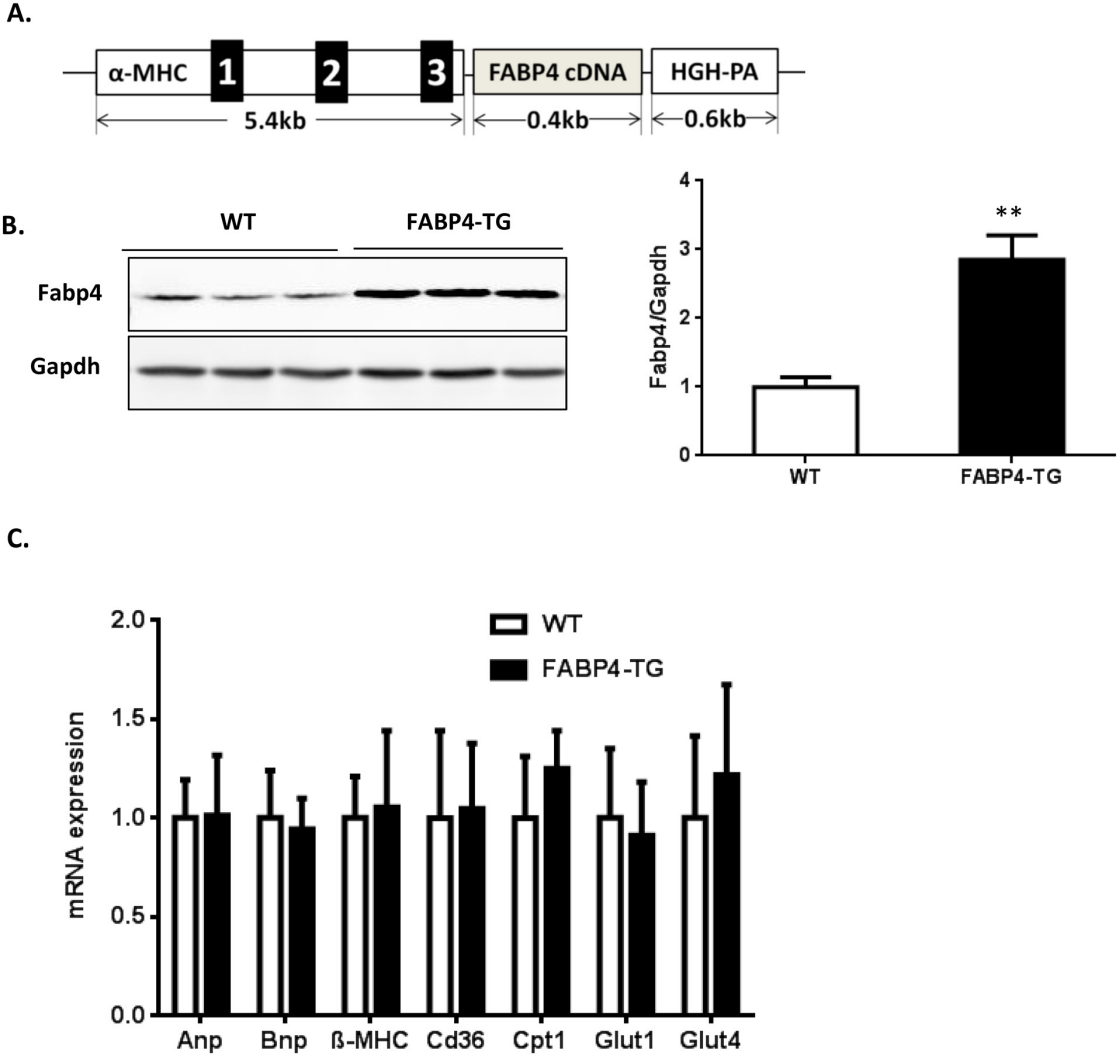
Generation of cardiomyocyte-specific FABP4 transgenic mice. (A) Schematics of mouse α-MHC-driven FABP4 transgenic construct, HGH-PA: human growth hormone polyadenylation signal. (B) The expression of FABP4 in isolated adult cardiomyocytes from wild type and FABP4-TG mice. Relative expression levels of FABP4 protein normalized by GAPDH are expressed as mean±SD (n = 6, **p<0.01) and shown in the right panel. (C) qRT-PCR was used to measure gene expression levels in hearts. (mean±SD, n = 8).

**Fig 3:**
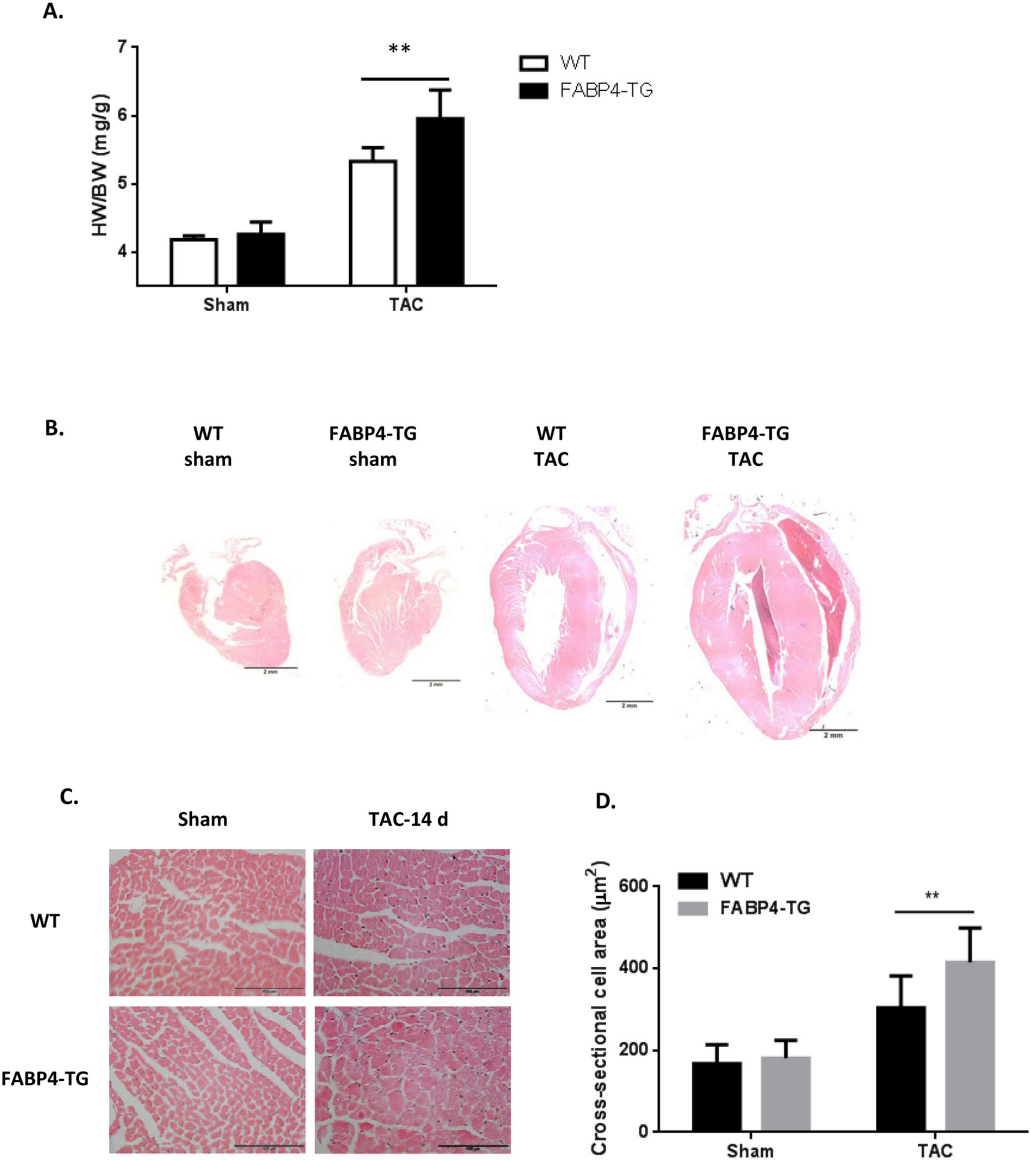
FABP4-TG mice have aggravated heart hypertrophy after TAC. (A) Heart weight to body weight ratio (HW/BW). Hearts were harvested at day 14 after TAC. (mean±SD, n = 7, **P<0.01). (B) Representative hematoxylin and eosin (H&E) stain of mice vertical heart sections of WT sham, FABP4-TG sham, WT TAC and FABP4-TG TAC. (C&D) H&E staining of left ventricular cross sections and the relative cross-sectional cell area in WT and FABP4-TG mice after TAC or sham operation.

**Fig 4:**
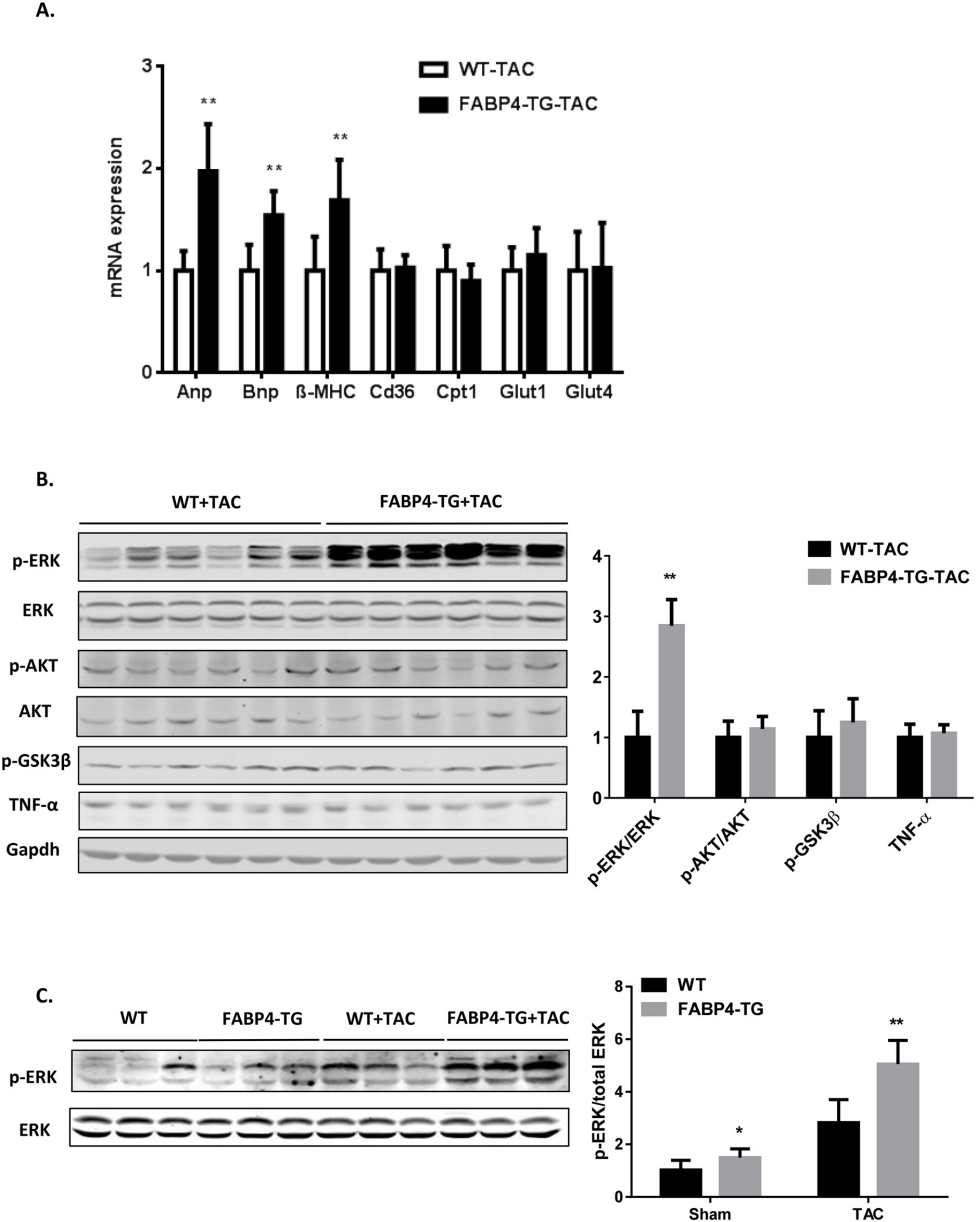
FABP4-TG mice have increased heart hypertrophy marker gene expressions and enhanced ERK phosphorylation after TAC. (A) qRT-PCR results showing increased heart hypertrophy marker gene expression levels in FABP4-TG TAC mice hearts (mean±SD, n = 7, **P<0.01). (B&C) Representative western blots showing increased phosphorylation of ERK signal in heart from FABP4-TG TAC compared with WT-TAC group (n = 6, *p<0.05, **p<0.01).

To define the roles of FABP4 in the heart and cardiomyocytes, we generated an α-MHC promoter derived human FABP4 transgenic mouse model with cardiac-specific FABP4 over-expression (Fig 2A) and two FABP4-TG mouse lines (line 1 and line 2) were created. The FABP4 over-expression was verified by Western blotting using isolated cardiomyocytes from FABP4-TG mice and their WT littermate controls (Fig 2B) as well as immunofluorescence (S2 Fig and Figure A in S3 Fig), which is about 3-fold increase in line 1 and 2.5-fold increase in line 2 FABP4-TG mice. We chose Line 1 of FABP4-TG mice for further investigations. At baseline, the FABP4-TG mice bred normally, and body weight, cardiac morphology and contractile function were similar to those non-transgenic mice. The key metabolic and hypertrophy gene expression levels in transgenic mice heart didn’t show significant differences with the WT littermates (Fig 2C). To induce cardiac hypertrophy, FABP4-TG mice and WT littermate control mice were subjected to TAC procedure. Two weeks after the surgery, both line1 and line 2 of FABP4-TG mice showed aggravated cardiac hypertrophy induced by pressure-overloading, as indicated by increased heart weight (HW)/body weight (BW) ratio (Fig 3A and Figure B in S3 Fig) compared with the WT controls. Histological examination of heart sections with H&E staining also presented a larger heart size and an increased cross-section area of cardiomyocytes in the FABP4-TG mice (Fig 3B and 3C). Consistent with these data, hearts from FABP4-TG mice showed greater hypertrophic marker (including ANP, BNP and β-MHC [34–36]) induction than control groups after TAC (Fig 4A). Echocardiograph data also confirmed that the TG mice have increased left ventricular internal diastolic diameter (LVIDd) and LV diastolic volume (Vol d) (Table 1). However, the cardiac function was not significantly changed as the major heart function indexes: LV fractional shortening (FS) and ejection function (EF) had no significant difference between the two groups (Table 1). We also assessed the effect of FABP4 over-expression on pressure overload induced cardiac fibrosis, and the sizes of fibrosis area didn’t show significant difference (S4 Fig).

**Table 1:** Characteristics and echocardiography measurements from WT and FABP4-TG mice 2 weeks after TAC or sham operation.

Groups	WT-Sham	FABP4-TG-Sham	WT-TAC	FABP4-TG-TAC
No. of Mice	8	8	7	8
BW (g)	24.7±2.4	24.9±1.4	24.8±1.0	26.4±2.1
HR (bpm)	458.5±68	450.6±51.7	494.6±38.8	460.8±51.8
LVIDd (mm)	3.7±0.2	3.6±0.2	3.7±0.2	4.0±0.1*
LVIDs (mm)	2.5±0.2	2.5±0.4	2.7±0.2	2.9±0.2
FS (%)	31.6±4.9	32.5±6.6	26.5±2.1	25.9±5.0
Vol d (mm^3^)	57.1±7.4	55.9±9.1	58.5±8.5	68.6±6.1*
Vol s (mm^3^)	22.8±4.7	22.2±8	27.8±5.2	33.5±5.7
SV (μl)	36.3±5.8	35.5±4.1	30.7±4.0	33.1±7.1
EF (%)	60.1±6.9	61.1±9.4	52.6±3.5	51.1±7.9

Data are represented as means±SD. TAC, transverse aorta constriction; BW, body weight; HR, heart rate; LVIDd, left ventricular internal diameter (diastole); LVIDs, left ventricular internal diameter (systole); FS, LV fractional shortening; Vol d, left ventricle volume diastole; Vol s, left ventricle volume systole; EF, LV ejection fraction.

*P <0.05 vs WT-TAC.

Cardiac hypertrophy is regulated by intracellular signaling pathways such as MAPK signaling and the PI3K/AKT pathway after initiation of signaling at the cell membrane [37, 38]. To gain insight into the molecular signal changes underlying the effect of FABP4 on aggravated cardiac hypertrophy, we examined the activation of MAPK/ERK signaling and PI3K-AKT pathway in heart following pressure overload induced by TAC. The phosphorylation of ERK1/2 was strongly induced in FABP4-TG mice (Fig 4B and 4C). However, p-AKT and GSK3β signals were not changed. Other inflammation signals like TNF-α were also undisturbed.

Taken together, these results indicated that increased FABP4 levels in cardiomyocytes aggravated the development of cardiac hypertrophy and activated ERK1/2 signaling pathway.

### FABP4 over-expression stimulates the phosphorylation of ERK in cardiomyocytes

**Fig 5:**
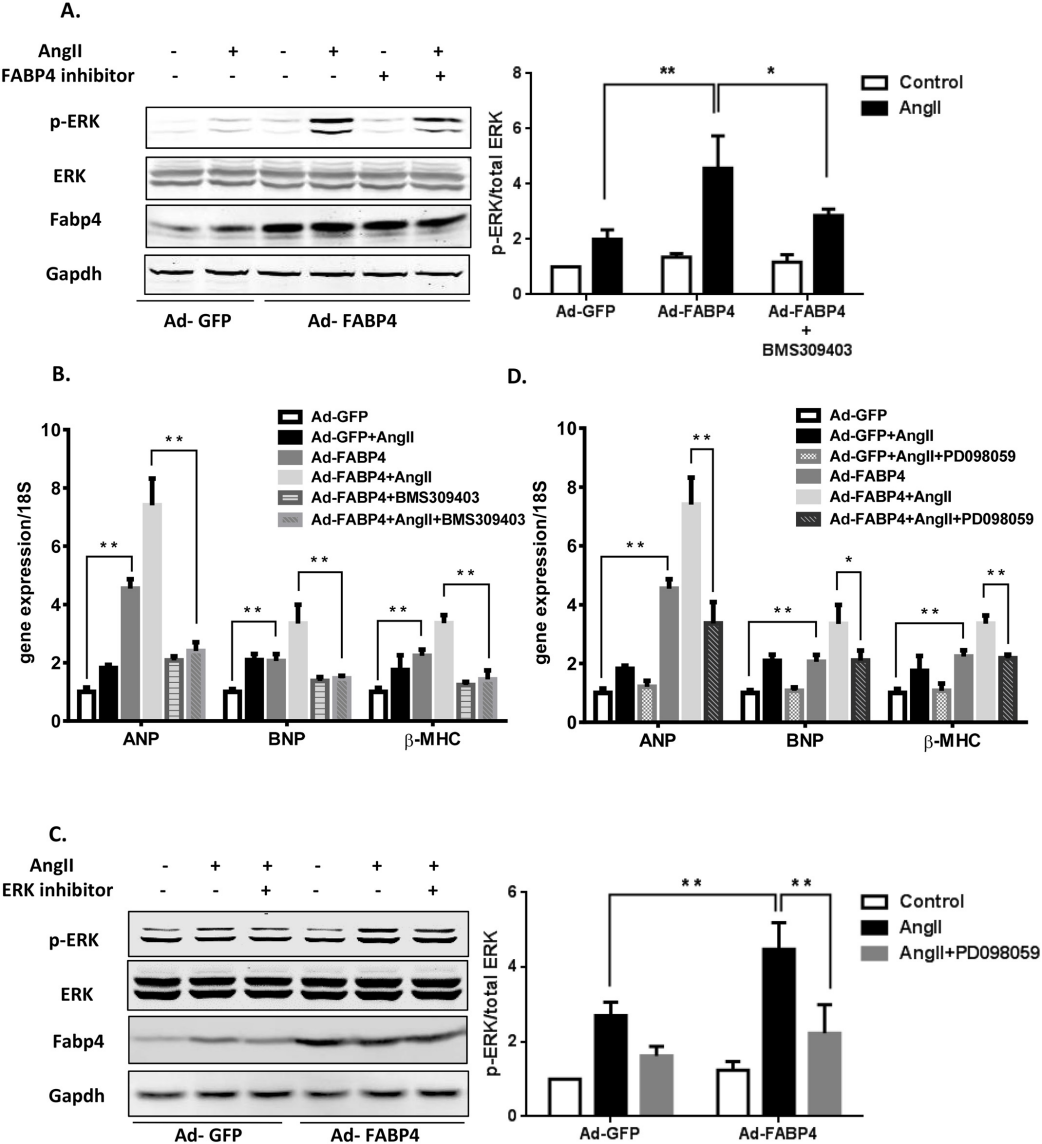
Involvement of ERK signaling in FABP4-promoted cardiac hypertrophy. Ad-FABP4 (Ad-GFP as control) infected NRCMs were pretreated with 25μM FABP4 inhibitor BMS309403 (A&B) or 50μM ERK inhibitor PD098059 (C&D) before 100nM Angiotensin II stimulation. (A&C) Cell lysates were prepared and subjected to western blotting analysis using antibodies against phospho-ERK1/2 and total ERK.; (B&D) Expressions of heart hypertrophy marker genes were measured by qRT-PCR. (n = 4, *p<0.05, **p<0.01)

To identify the molecular mechanism of FABP4 induced cardiac hypertrophy, we performed *in vitro* studies using primary cultured NRCMs. NCRMs were infected with Ad-FABP4 to over-express FABP4 (Ad-GFP as control) and subsequently exposed to cardiac hypertrophy agonist angiotensin II (Ang II) [39, 40]. Consistent with the *in vivo* data, ERK phosphorylation was strongly enhanced by FABP4 over-expression (Fig 5A). Notably, the Ang II-induced expression of hypertrophic hall marks (ANP, BNP and β-MHC) were also profoundly enhanced in Ad-FABP4-infected NRCMs compared with controls (Fig 5B). BMS309403 is a small molecule and has been used as FABP4 inhibitor [17, 41]. By pre-treating NRCMs with 25μM of BMS309403 for 1 hour before the Ang II stimulation, the FABP4 enhanced ERK activation and induced hypertrophic marker gene expressions can be significantly repressed (Fig 5A and 5B). These results further revealed that FABP4 had a pro-hypertrophic effect in cardiomyocytes.

### ERK inhibitor attenuated the signal in FABP4 induced cardiac hypertrophy

We next sought to test whether ERK1/2 signaling activation is required for FABP4 mediated induction of cardiac hypertrophy. The effects of FABP4 on cardiac hypertrophy were investigated in the presence of an ERK signaling inhibitor PD098059 [42]. NRCMs were incubated with 50μM PD098059 for 1 hour before treatment with 100nM Ang II. Cells were then prepared and subjected to Western blotting using phosphorylated ERK antibodies. Similar with the results in Fig 4A, the strongly phosphorylated ERK signal in FABP4 over-expressed NRCMs can be blocked by ERK inhibitor treatment (Fig 5C). After analyzing the hypertrophic marker gene mRNA levels by qRT-PCR, we found that the expression of ANP, BNP and β-MHC were prohibited by PD098059 pre-treatment in FABP4 over-expressed NRCMs (Fig 5D). *In vitro* data also demonstrated that Ang II induced cardiomyocytes hypertrophy was enhanced in Ad-FABP4-infected NRCMs. Furthermore, this cell size enlargement of cardiomyocyte by Ang II and FABP4 over-expression can also be attenuated by PD098059, as shown in S5 Fig. Taken together, these data suggest that the ERK activation is essential for FABP4 induced cardiac hypertrophy.

## Discussion

In the present study, we demonstrated several significant findings. (1) FABP4 is expressed in cardiomyocytes and can be up-regulated by PPARγ activation; (2) Cardiomyocyte-specific FABP4 mice developed aggravated cardiac hypertrophy under pressure overload; (3) FABP4 over-expression in cardiomyocyte activates ERK phosphorylation, and ERK inhibitor can abolish FABP4 induced cardiomyocytes hypertrophy effect. These findings demonstrated that FABP4 in cardiomyocytes involves the development of cardiac hypertrophy through ERK activation.

FABP4 has long been known to play a significant role in metabolic syndrome and the function of FABP4 in cardiovascular system has drawn increasing attention in recent years. Previous studies indicated that circulating FABP4 level is an independent cardiac risk marker, and exogenous FABP4, secreted by adipose tissue or macrophage, can suppress cardiomyocytes contraction and may directly regulate cardiac function [5, 14, 43–46]. Although FABP4 is closely associated with cardiac dysfunction, the role of endogenous FABP4 in heart especially in cardiomyocytes has never been investigated. Our study demonstrated for the first time that FABP4 plays a pro-hypertrophic role in cardiomyocytes. Heart has abundant FABP4 expression, but the capillary endothelial cell has been considered the main source previously. In our prelim experiments, we found that FABP4 was also expressed in cardiomyocytes and could be strongly induced by PPARγ over-expression as well as PPARγ agonists treatment, suggesting that FABP4 may have important roles in cardiomyocytes. Considering that the related cardiac risk of using PPARγ agonists as anti-diabetic drugs is still under debate [47, 48], it is of potential importance to study the functions of this PPARγ target gene FABP4 in heart and cardiomyocytes.

Our cardiomyocyte-specific FABP4 over-expression transgenic mice had normal phenotype at baseline. Under TAC induced pressure overload condition, the transgenic mice have increased left ventricle internal diastolic dimension (LVIDd), left ventricle volume diastole (Vol d) and developed more cardiac hypertrophy than WT littermate controls. Meanwhile, those animals still maintain similar heart functions, as LV fractional shortening (FS) and ejection function (EF) had no significant difference with the WT group, indicating that the animals are able to compensate the aggravated hypertrophy effect caused by FABP4 in our TAC model.

To identify the mechanisms underlying the FABP4 induced cardiac hypertrophy, we examined those well characterized hypertrophy signal pathways, and our result showed that ERK signaling are strongly induced in FABP4-TG mice heart. These results were also confirmed in isolated neonatal cardiomyocytes using angiotensin II treatment in the presence of ERK inhibitor or FABP4 inhibitor. Both of these two inhibitors abolished the enhanced p-ERK signals in FABP4 over-expression cells and inhibited the relative increased expressions of hypertrophy marker genes (ANP, BNP and β-MHC). ERK1/2 has been considered as the central mediator of cardiac hypertrophy, although the therapeutic value of inhibiting ERK in pressure overload induced cardiac hypertrophy remained disputable [49], previous investigations have clearly demonstrated that cardiac ERK1/2 over-activation directly leads to heart hypertrophy [50]. Our data here suggest that the ERK1/2 pathway inhibitor PD98059, as well as FABP4 inhibitor BMS309403, significantly attenuated the cardiomyocyte hypertrophy induced by FABP4 over-expression *in vitro*.

The detail mechanisms underlying FABP4’s multiple functions are largely unknown due to its complexity [51]. As a lipid carrier, it directly regulates intracellular free fatty acid levels and may indirectly control multiple gene expression involving inflammation and ER stress [52]. Exogenous FABP4 also have many important functions in obesity and atherogenesis. It directly suppresses cardiomyocyte contraction and possibly plays an important role in heart diseases. Our results demonstrated for the first time that FABP4 could enhance ERK activation in cardiomyocytes. This enhancement may depend on multiple pathways. Our preliminary data showed that the Ang II receptors (AT1a and AT2, data not shown) expressions were upregulated in FABP4 over-expressing cardiomyocytes by qRT-PCR. This up-regulation may increase the cellular response to Ang II stimulation and enhance downstream ERK phosphorylation. The exogenous FABP4 can also contribute to this process. As a secreted protein, over-expressing FABP4 in cardiomyocytes will elevate the soluble FABP4 in medium. We also found that extracellular FABP4 can directly activate ERK phosphorylation. Thus, the FABP4 secreted by cardiomyocytes may activate ERK and related hypertrophy signals through an autocrine/paracrine manner. However, due to the limited knowledge about the possible FABP4 receptor on the cell membrane, we cannot make a final conclusion at this time. These hypothesis and detail mechanism need further investigation in the future.

In conclusion, our data demonstrated for the first time that FABP4 expressed in the cardiomyocytes and can promote cardiac hypertrophy by activating ERK signal. It is therefore that inhibition of FABP4 in human might show beneficial effects against heart hypertrophy and cardiac events associated with PPARγ agonist drugs.

## Supporting Information

S1 Fig FABP4 is expressed in cardiomyocyte. (A) The expression of FABP4 in cardiomyocyte was examined by immunofluorescence using FABP4 antibody. Increased FABP4 signal can be detected in NRCM after Rosiglitazone treatment (1μM, 24 hours). (B) The expression of FABP4 in cardiomyocyte compared with cardiac fibroblast was shown in western blot. C. The expression of FABP4 in WT mice heart compared with FABP4 KO mice heart was examined by western blot, showing the specificity of FABP4 antibody.
(TIF)

S2 Fig FABP4 over-expression in WT and FABP4-TG mice heart. Representative immunofluorescence (anti-FABP4) of heart sections were shown as indicated.
(TIF)

S3 Fig Characterization of FABP4-TG mice line 2. (A) The expression of FABP4 in isolated adult cardiomyocytes from wild type and FABP4-TG line 2 mice. (B) Heart weight to body weight ratio (HW/BW) of FABP4-TG mice line 2. Hearts were harvested at day 14 after TAC. (mean±SD, n = 6–7, *P<0.05).
(TIF)

S4 Fig Cadiac Fibrosis comparison. Representative Sirius Red stain of mice vertical heart sections of WT TAC and FABP4-TG TAC.
(TIF)

S5 Fig FABP4 over-expression in cardiomyocyte enhances Ang II-induced hypertrophy. Ad-FABP4 (Ad-GFP as control) infected NRCMs were pretreated with 50μM ERK inhibitor PD098059 before 100nM angiotensin II stimulation. Representative immunofluorescence (anti-Troponin I) of cardiomyocytes were shown as indicated. The cell surface area was analyzed using ImageJ and statistical difference was shown. (n = 14, mean±SD from. *P < 0.05, **P < 0.01)
(TIF)

S1 Table Primer list. (DOCX)
