# Supplementary figures and images for: Cardiomyocyte Overexpression of FABP4 Aggravates Pressure Overload-Induced Heart Hypertrophy
Source: PLoS One. 2016 Jun 13;11(6):e0157372. doi: 10.1371/journal.pone.0157372 (PMC4905683; doi:10.1371/journal.pone.0157372)

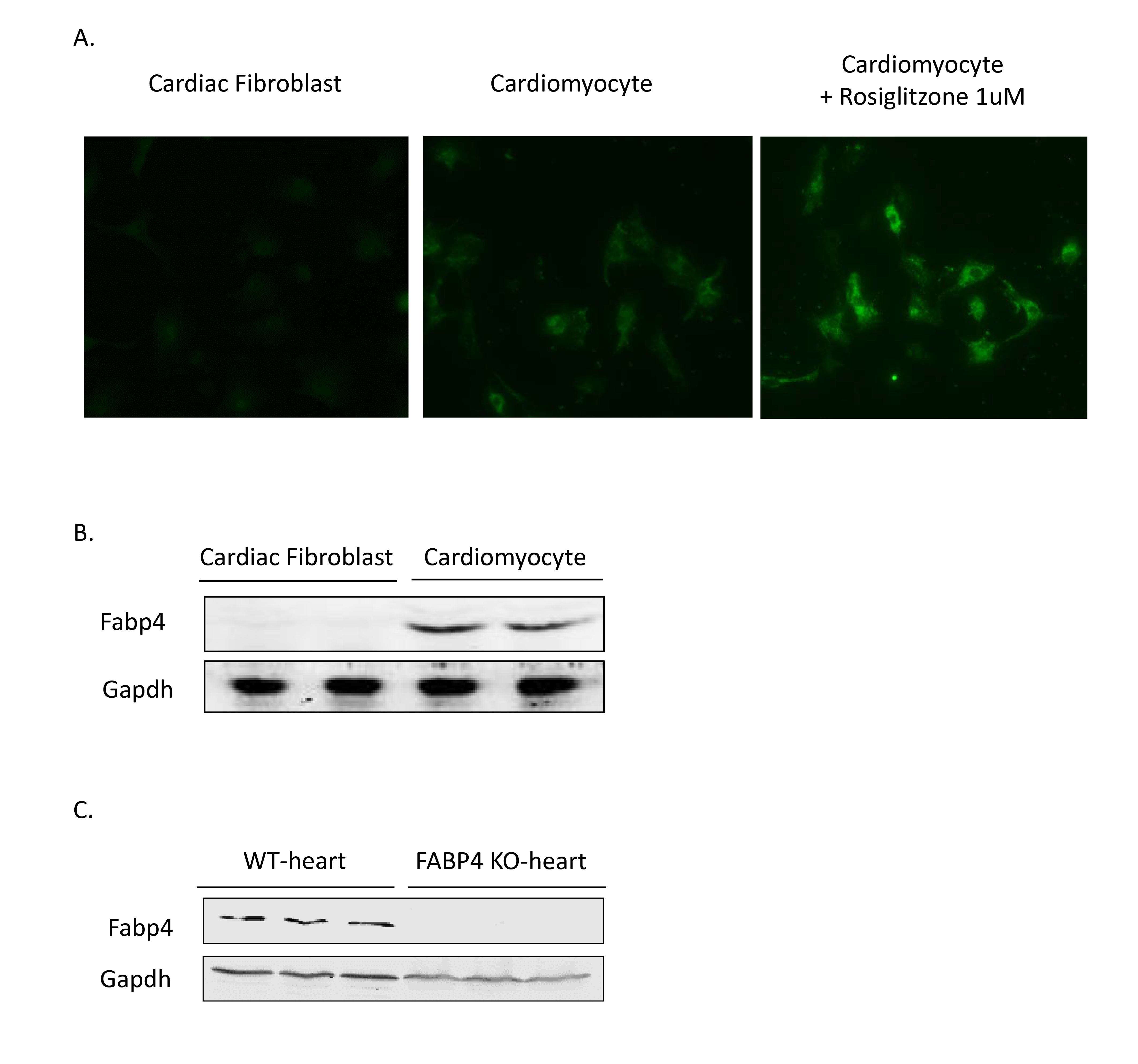

Supplement: S1 Fig — (A) The expression of FABP4 in cardiomyocyte was examined by immunofluorescence using FABP4 antibody. Increased FABP4 signal can be detected in NRCM after Rosiglitazone treatment (1μM, 24 hours). (B) The expression of FABP4 in cardiomyocyte compared with cardiac fibroblast was shown in western blot. C. The expression of FABP4 in WT mice heart compared with FABP4 KO mice heart was examined by western blot, showing the specificity of FABP4 antibody. (TIF) [file pone.0157372.s001.tif]

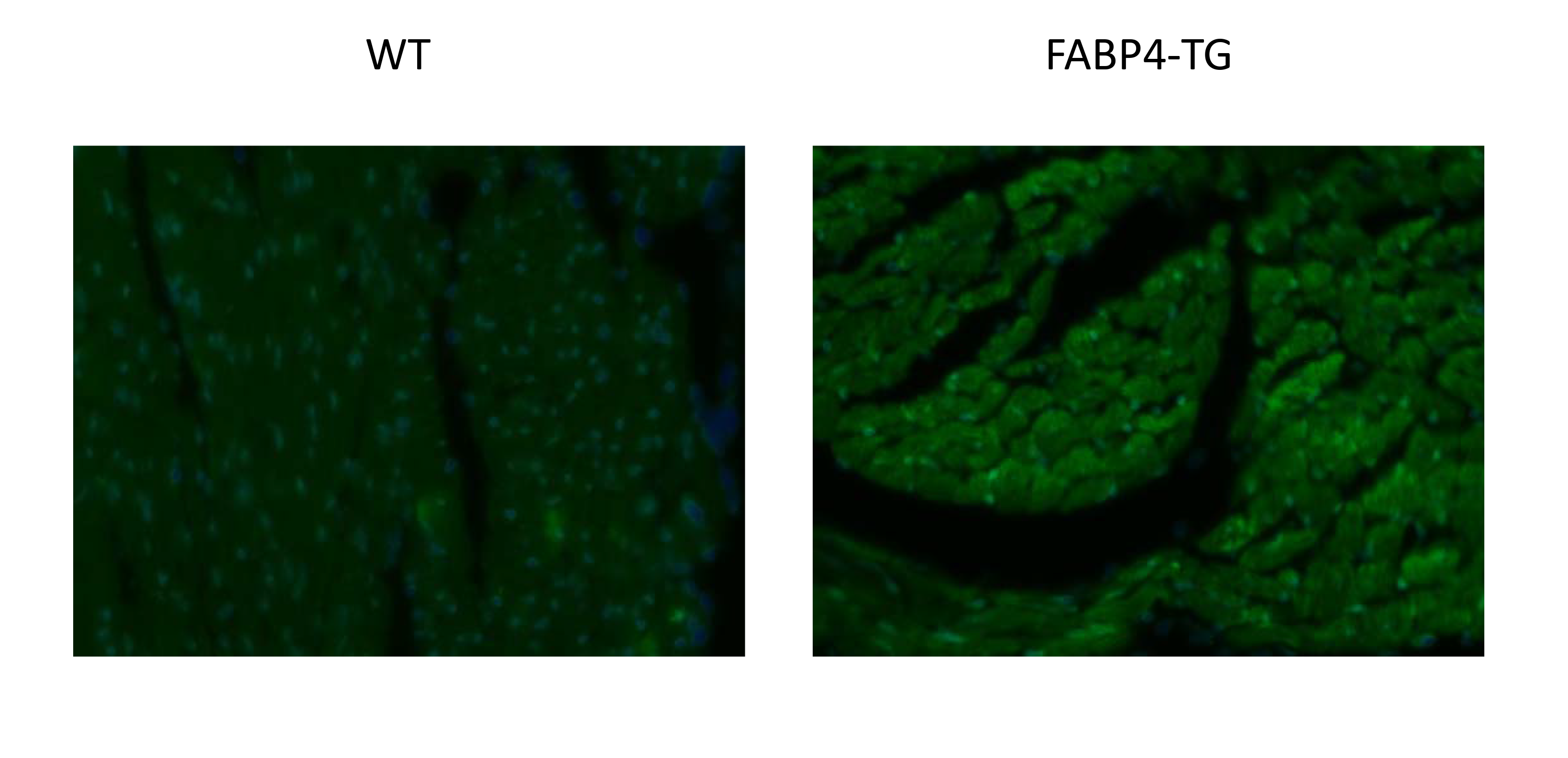

Supplement: S2 Fig — Representative immunofluorescence (anti-FABP4) of heart sections were shown as indicated. (TIF) [file pone.0157372.s002.tif]

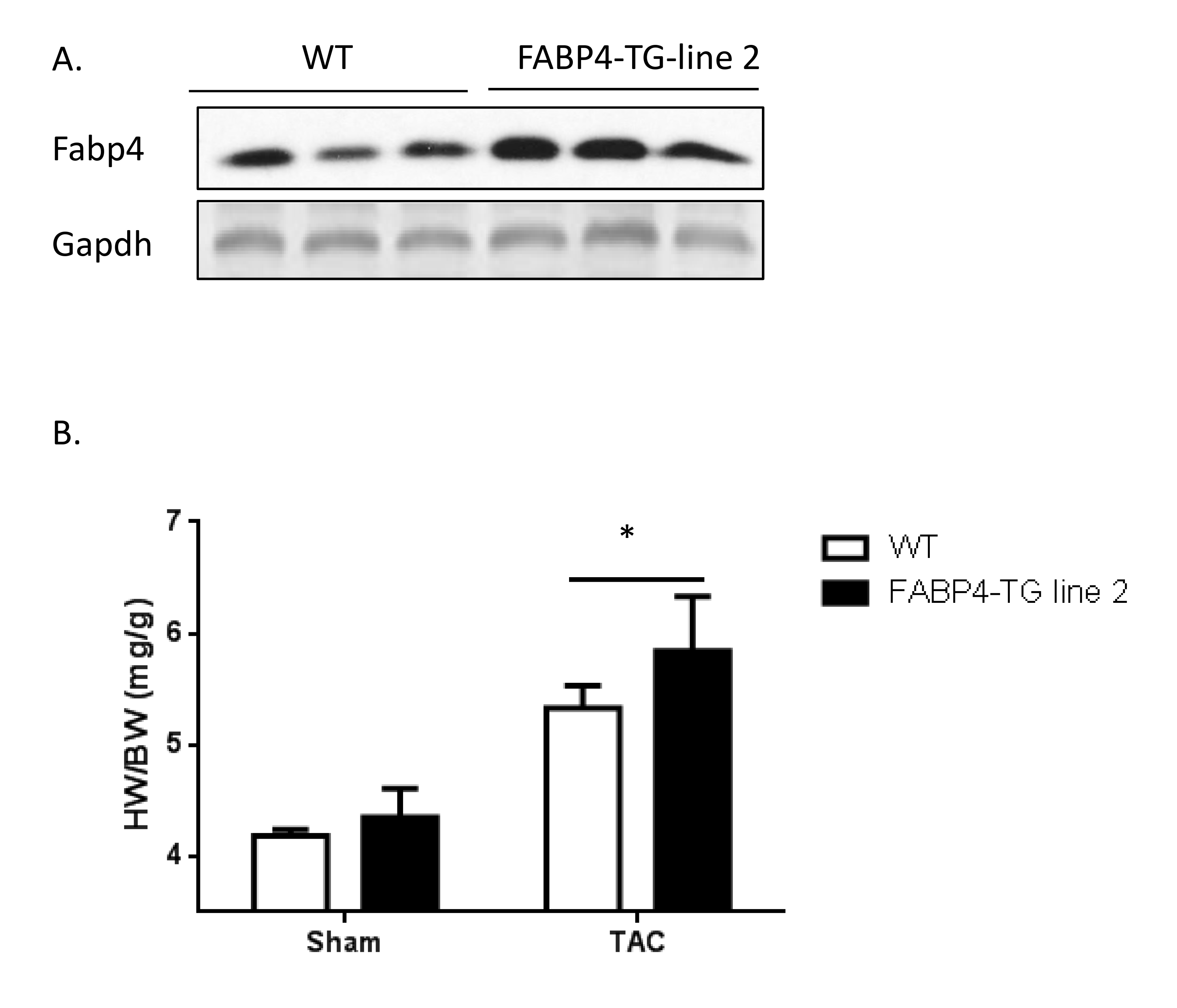

Supplement: S3 Fig — (A) The expression of FABP4 in isolated adult cardiomyocytes from wild type and FABP4-TG line 2 mice. (B) Heart weight to body weight ratio (HW/BW) of FABP4-TG mice line 2. Hearts were harvested at day 14 after TAC. (mean±SD, n = 6–7, *P<0.05). (TIF) [file pone.0157372.s003.tif]

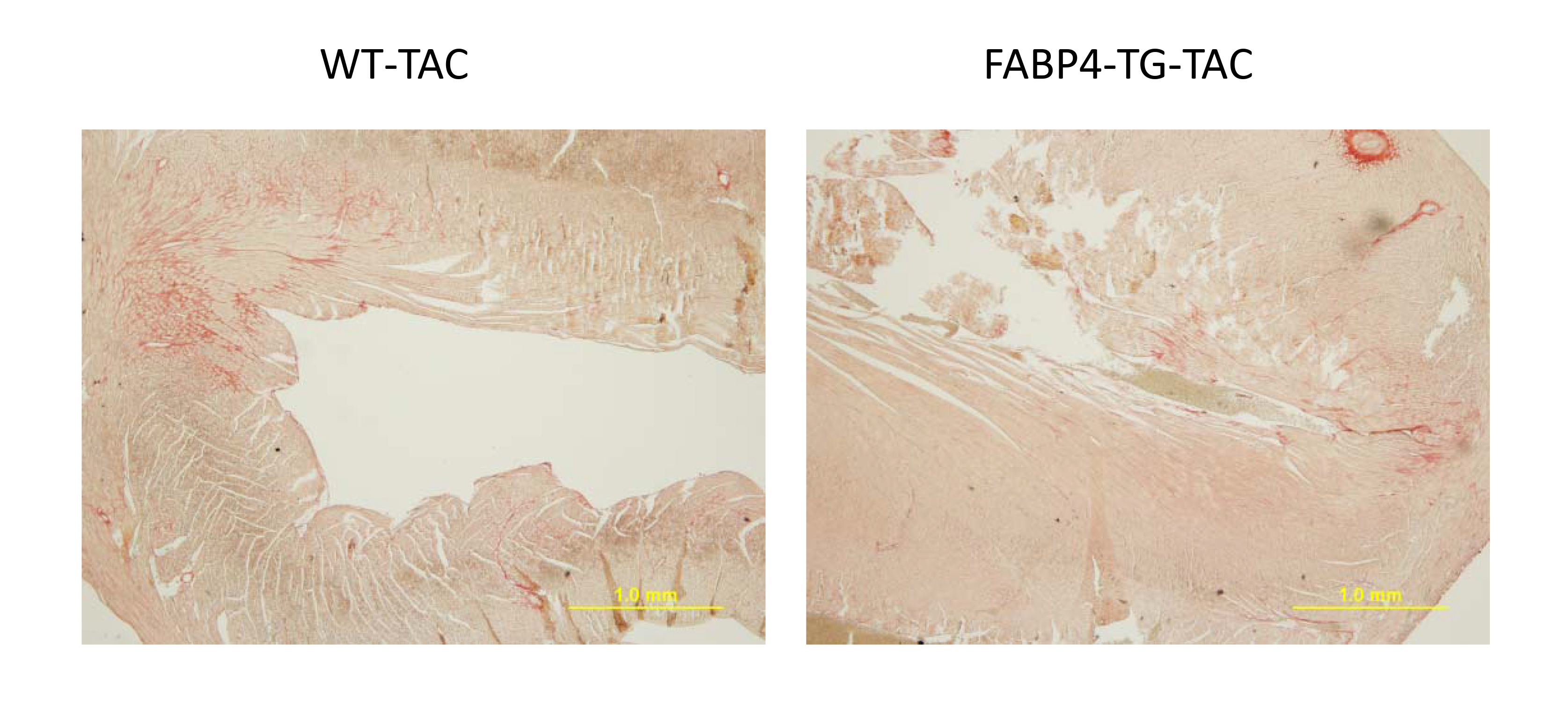

Supplement: S4 Fig — Representative Sirius Red stain of mice vertical heart sections of WT TAC and FABP4-TG TAC. (TIF) [file pone.0157372.s004.tif]

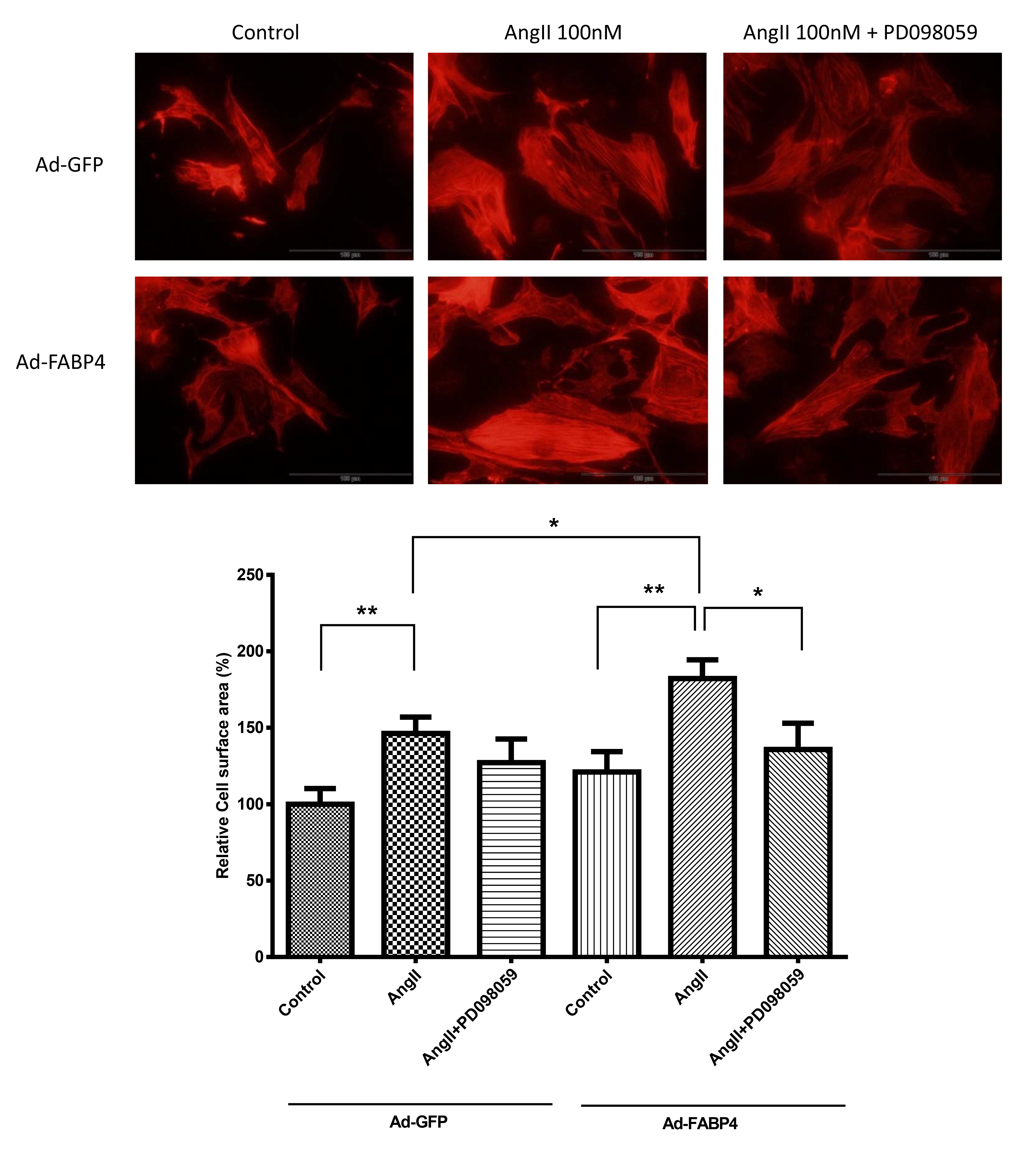

Supplement: S5 Fig — Ad-FABP4 (Ad-GFP as control) infected NRCMs were pretreated with 50μM ERK inhibitor PD098059 before 100nM angiotensin II stimulation. Representative immunofluorescence (anti-Troponin I) of cardiomyocytes were shown as indicated. The cell surface area was analyzed using ImageJ and statistical difference was shown. (n = 14, mean±SD from. *P < 0.05, **P < 0.01) (TIF) [file pone.0157372.s005.tif]
