# Supplementary material for: Cardiomyocyte Overexpression of FABP4 Aggravates Pressure Overload-Induced Heart Hypertrophy
Source: PLoS One. 2016 Jun 13;11(6):e0157372. doi: 10.1371/journal.pone.0157372 (PMC4905683; doi:10.1371/journal.pone.0157372)
Supplement: S1 Table — (DOCX) [file pone.0157372.s006.docx]

| Gene | Primers |
| --- | --- |
|  | (5’-3’) |
| mouse Anp F | GCTTCCAGGCCATATTGGAG |
| mouse Anp R | GGGGGCATGACCTCATCTT |
|  |  |
| mouse Bnp F | GAGGTCACTCCTATCCTCTGG |
| mouse Bnp R | GCCATTTCCTCCGACTTTTCTC |
|  |  |
| mouse β-MHC F | ACTGTCAACACTAAGAGGGTCA |
| mouse β-MHC R | TTGGATGATTTGATCTTCCAGGG |
|  |  |
| mouse Fabp4 F | AAGGTGAAGAGCATCATAACCCT |
| mouse Fabp4 R | TCACGCCTTTCATAACACATTCC |
|  |  |
| mouse Cd36 F | ATGGGCTGTGATCGGAACTG |
| mouse Cd36 R | TTTGCCACGTCATCTGGGTTT |
|  |  |
| mouse Cpt1 F | GACTTCCGGCTTAGTCGGG |
| mouse Cpt1 R | GAATAAGGCGTTTCTTCCAGGA |
|  |  |
| mouse Glut1 F | GCAGTTCGGCTATAACACTGG |
| mouse Glut1 R | GCGGTGGTTCCATGTTTGATTG |
|  |  |
| mouse Glut4 F | ACACTGGTCCTAGCTGTATTCT |
| mouse Glut4 R | CCAGCCACGTTGCATTGTA |
|  |  |
| rat Anp F | GGGGGTAGGATTGACAGGAT |
| rat Anp R | CTCCAGGAGGGTATTCACCA |
|  |  |
| rat Bnp F | GACGGGCTGAGGTTGTTTTA |
| rat Bnp R | ACTGTGGCAAGTTTGTGCTG |
|  |  |
| rat β-MHC F | CCTCGCAATATCAAGGGAAA |
| rat β-MHCR | TACAGGTGCATCAGCTCCAG |
